# Bridging the immunisation gap: socioeconomic and geographic drivers of pediatric immunisation disparities between different Indian states

**DOI:** 10.1186/s12887-026-06822-6

**Published:** 2026-04-14

**Authors:** Yendouname Kandjoni, Samadou Tchakondo, Ayao Sangénis Assogba, Komi Selassi Gayi, Richard Sagacity Tugbeh, Kezia Angeline J, Gladius Jennifer Hirudayaraj

**Affiliations:** 1 SRM School of Public Health, Faculty of Medicine and Health Sciences, SRM Institute of Science and Technology, Kattankulathur Campus, Chengalpattu, Chennai, 603203 India; 2 National School of Medical Auxiliaries (ENAM), Faculty of Health Sciences, University of Lomé, 1012 Boulevard de la victoire, Lomé, Togo; 3 Higher School of Biological and Food Techniques (ESTBA), Faculty of Health Sciences, University of Lomé, Lomé, Togo; 4 Laboratory of Biomedical, Food and Environmental Health Sciences- Biomedical Sciences and Bioactive Substances Research Unit (LaSBASE-UR- 2SB), University of Lomé, Lomé, Togo

**Keywords:** Immunisation, Socioeconomic determinants, Geographic drivers, India, Maternal education, Health disparities

## Abstract

**Background:**

Immunising children saves 2–3 million lives every year, making it one of the most effective public health interventions. India’s childhood immunisation rates are steadily improving, but there are still significant differences between states. Tamil Nadu consistently has one of the highest rates of child immunisation, while Nagaland is below the national average. If we figure out the socioeconomic factors that contribute to this difference, we can guide context-specific strategies to improve coverage.

**Objective:**

To compare the level and socioeconomic and geographic determinants of full immunisation coverage among children aged 12–23 months in Tamil Nadu and Nagaland using data from the fifth National Family Health Survey (NFHS-5, 2019-21).

**Methods:**

A survey-weighted cross-sectional analysis was performed on 1851 children (Tamil Nadu: 1291; Nagaland: 560). To be fully immunised, a child had to receive BCG, three doses of DPT, three doses of OPV, and one dose of MCV. Weighted bivariate chi-square tests and multivariable logistic regression models were used to assess associations between full Immunisation and socioeconomic, demographic, and geographic characteristics. We performed sensitivity analysis by restricting to only children who possessed vaccination cards, to assess the robustness of our findings.

**Results:**

Immunisation coverage was higher in Tamil Nadu (89.2%) than in Nagaland (57.9%). In bivariate analysis, maternal education was significantly associated with full Immunisation in both states, while wealth, residence, distance to health facility, and birth order were additionally significant in Nagaland, and residence and religion in Tamil Nadu.In multivariable analysis, maternal education remained the most consistent predictor in Nagaland (AOR=2.61; p=0.003) and Tamil Nadu (AOR=2.32; p=0.029). In Nagaland, distance to health facility was also significant (p=0.034), whereas in Tamil Nadu, urban residence (AOR=1.83; p=0.008) and religion (AOR=4.00; p=0.003) were associated with Immunisation status. Other variables were not significant after adjustment.

**Conclusion:**

The main factors for the differences in immunisation coverage between states are gaps in maternal education and access to healthcare. To increase vaccination coverage in states with lower coverage, it is crucial to enhance women's education, extend outreach to rural populations, and address socioeconomic barriers.

**Supplementary Information:**

The online version contains supplementary material available at 10.1186/s12887-026-06822-6.

## Introduction

Childhood Immunisation is still one of the most effective public health measures, saving an estimated two to three million lives each year against vaccine-preventable diseases [1, 2]. The Expanded Programme on Immunisation (EPI), which the World Health Organisation established in 1974, aimed to make sure that every child received essential vaccines that can save them against common childhood diseases like measles, polio, diphtheria, pertussis, tetanus, and tuberculosis [1].

In India, the history of organized vaccination finds its origin back in approximately two centuries. The first documented inoculation took place in Bombay (now Mumbai) in 1802, marking the beginning of a long national engagement with preventive medicine [3]. After independence, India invested heavily and put significant effort into national vaccine production and distribution. The government introduced the Expanded Programme of Immunisation in 1978, followed by the Universal Immunisation Programme (UIP) in 1985, which remains one of the largest Immunisation initiatives in the world [4].

Although national efforts like Intensified Mission Indradhanush (IMI) [18] have considerably improved vaccine coverage, significant differences persist between states. India’s average full Immunisation rate was reported at 93.5% in 2023-24, yet coverage remains uneven across states, for instance Tamil Nadu achieved roughly 90%, while Nagaland lagged at about 58% [5–6]. These differences show that there are differences in access including geographic terrain, infrastructure, and social factors that affect child health outcomes in different areas.

Apart from vaccine availability, accessibility, affordability, studies consistently show that socioeconomic and geographic factors play a decisive role in Immunisation services utilization. Maternal education, in particular, enhances awareness of preventive care and the ability to navigate health services. In India, maternal education and child immunisation have been shown to be strongly positively correlated by Vikram et al. (2012) and by Sinha et al. (2020) [7, 9], with regional and gender differences also being reported. Sinha et al. (2013) found that children living in rural areas and female children were less likely to be fully Immunised [8].

But even with all these revelations, there hasn’t been much comparative research done on why some states for example Tamil Nadu, maintain nearly universal coverage, while others like Nagaland continue to lag behind. It is then important to figure out these inequalities in order to apply effective models to settings with comparatively lower coverage.

Following all of these, our study utilizes data from the fifth National Family Health Survey (NFHS-5, 2019-21) to compare the socioeconomic determinants of full immunisation in Tamil Nadu and Nagaland. We intend through this study to effectively inform evidence-based interventions that support an equitable and long-term immunisation coverage throughout India by identifying state-specific predictors and transferable strategies.

## Methodology

### Study design, data source and extraction

This study was a cross-sectional analytical design, we used publicly available secondary data from the National Family Health Survey (NFHS-5, 2019–2021). It was conducted by the International Institute for Population Sciences (IIPS) in collaboration with the Ministry of Health and Family Welfare, Government of India, NFHS-5 and provides nationally representative data on key demographic and health indicators [10].

The analysis used the NFHS-5 children’s recode file obtained from the DHS Program after authorization. From that file, we extracted records for children aged 12–23 months and limited the dataset to observations from the two states of interest (Tamil Nadu and Nagaland). For state-specific comparisons we created two separate datasets by applying the state filter to the extracted child records (one dataset for Tamil Nadu, one for Nagaland) and retained a pooled dataset for overall descriptive reporting.

### Study population

We included children aged 12–23 months residing in the Indian states of Tamil Nadu and Nagaland, consistent with the standard age group used for assessing full immunisation coverage in Demographic and Health Surveys (DHS). Children outside this age range were excluded.

**Fig. 1:**
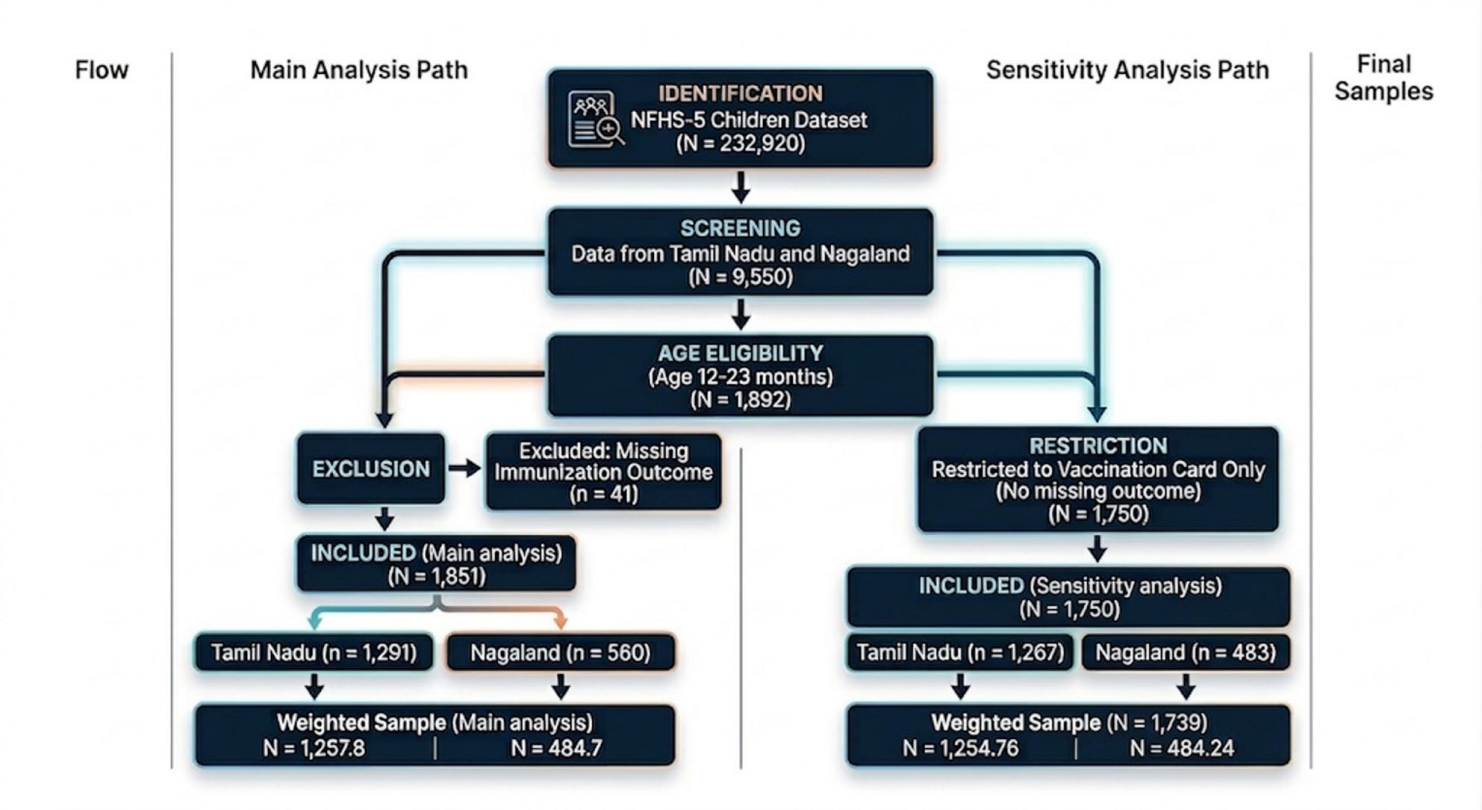
Diagram of sample selection flow for the main and sensitivity analysis of the NFHS-5 children dataset

For the primary analysis, all eligible children with valid immunisation information (based on vaccination card and/or mother’s recall) were included. After applying these criteria, the final analytic sample comprised 1851 children (Tamil Nadu: *n* = 1291; Nagaland: *n* = 560) (Fig. 1).

A secondary, restricted sample consisting only of children with available vaccination cards was used for sensitivity analysis. 142 children were excluded due to lack of cards. The final restricted weighted sample included 1,739 children (Tamil Nadu: *n* = 1,255; Nagaland: *n* = 484) (Fig. 1).

### Variable definition

#### Outcome variable

Our primary outcome, full immunisation status, was defined according to standard WHO and DHS guidelines, combining information from vaccination cards and maternal recall. A child was classified as “fully immunised” (1) if they had received one dose of Bacillus Calmette-Guérin (BCG), three doses of diphtheria-pertussis-tetanus (DPT), three doses of oral polio vaccine (OPV), and one dose of a measles-containing vaccine (MCV). We classified as “not fully immunised” (0), children missing any of these doses.

For sensitivity analysis, we redefined full immunisation using vaccination card records only, excluding maternal recall. This approach was used to assess the robustness of findings and the potential impact of recall bias; however, given that vaccination card possession is associated with socioeconomic and healthcare access factors, this restricted definition may introduce selection bias and was therefore not used as the primary analytical approach.

#### Explanatory variables

Our independent variables included maternal age, maternal education, household wealth index, place of residence, religion, perceived distance to health facility, birth order, and sex of the child.

All variables were coded and harmonized according to DHS standard definitions and analytical recoding procedures as shown in Table 1.

**Table 1:** Independent variables and their categories

Variable	Categories / Coding
Maternal education	No education, Primary, Secondary, Higher
Wealth index	Poor, Middle, Rich [17]
Place of residence	Urban, Rural
Religion	Hindu, Others
Birth order	1, 2, ≥ 3.
Distance to health facility	No problem, Big problem
Sex of the child	Male, Female
Mother’s age	Continuous and grouped (18–24, 25–34, ≥ 35 years)

### Data weighting and missing data handling

We applied sampling weights to ensure representativeness of state-level estimates. The DHS child sampling weight (v005) was divided by 1,000,000 and applied in all descriptive and inferential analyses. The survey design was accounted for using primary sampling units (clusters) and stratification variables provided in the DHS dataset (V021 and V022). We excluded listwise, missing data on Immunisation status (2.2% which is less than 5% of observations). Independent variables showed no missing values in our sample size. We have cleaned and recoded the data in IBM SPSS Statistics Version 25.

### Statistical analysis and software

We performed weighted descriptive statistics to summarize the distribution of child immunisation, maternal, and household characteristics. Rao-Scott adjusted chi-square (χ²) tests assessed bivariate associations between Immunisation status and explanatory variables.

To identify independent predictors, binary logistic regression models were fitted separately for Tamil Nadu and Nagaland, adjusting for all covariates. We expressed the results as Adjusted Odds Ratios (AORs) with 95% Confidence Intervals (CIs) and p-values. We assessed multicollinearity using the Variance Inflation Factor (VIF < 2) and Tolerance (> 0.1). We did not detect any multicollinearity or influential outliers (Supplementary Table 3).

The regression model was specified as:

$$\:\mathrm{logit}\text{}\left({p}_{i}\right)={\beta\:}_{0}+\sum\:{\beta\:}_{k}{X}_{ki}$$

where $\:{p}_{i}\:$is the probability of being fully Immunised, and $\:{X}_{ki}\:$represents predictor variables.

All analyses were stratified by state to facilitate cross-state comparison of determinants and were conducted in IBM SPSS Statistics 25, and statistical significance was determined at *p* < 0.05 (two-tailed).

### Ethical considerations

We used anonymized, publicly available NFHS-5 data and therefore did not require ethical approval. The DHS Program obtained informed consent from all respondents during primary data collection. We have used the data in compliance with the DHS terms and conditions.

## Results

### Characteristics of the study population

**Fig. 2:**
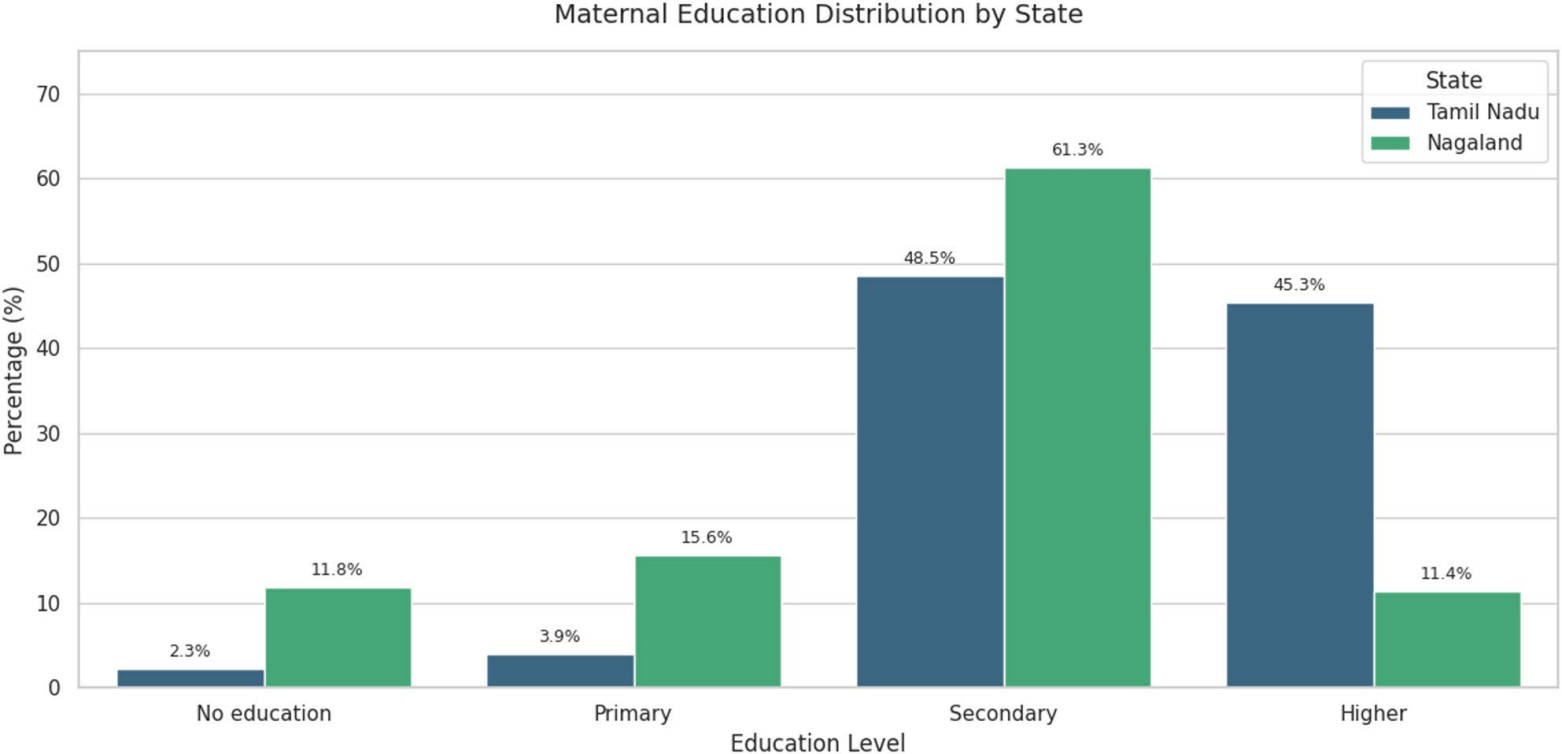
Mother’s education level distribution per states

A total of 1851 children aged 12–23 months were included in the analysis; 1291 from Tamil Nadu and 560 from Nagaland. The mean age of mothers was 26.56 years in Tamil Nadu and 28.74 years in Nagaland. More than 52% of children in Tamil Nadu resided in rural areas, compared with 80.5% in Nagaland. Educational attainment differed substantially between the two states, 93.8% of mothers in Tamil Nadu had at least secondary education, whereas only 72.7% of mothers in Nagaland did (Fig. 2).

**Fig. 3:**
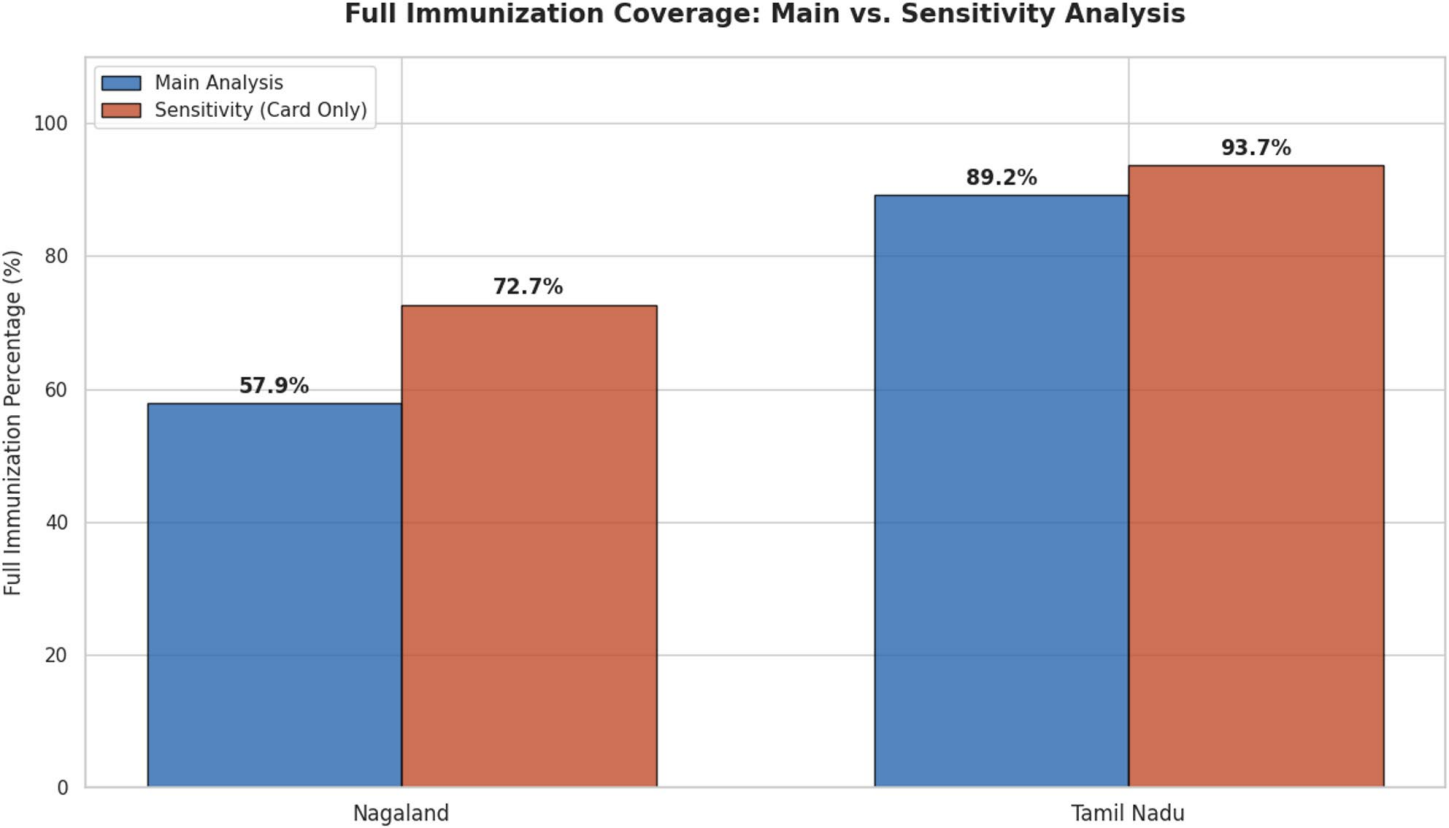
Immunisation coverage distribution by States (Main and sensitivity analyses)

Full Immunisation coverage based on the standard DHS definition (vaccination card or mother’s recall) was 89.2% in Tamil Nadu and 57.9% in Nagaland, confirming the substantial interstate disparity identified in the NFHS-5 dataset (Fig. 3). In sensitivity analyses restricted to children with vaccination cards, coverage estimates were higher but still showing substantial interstate disparity, at 93.7% in Tamil Nadu and 72.7% in Nagaland (Fig. 3). These high coverages in the sensitivity analysis reflect the potential influence of selection bias associated with card availability.

### Factors associated with immunisation coverage in Tamil Nadu and Nagaland

Table 2 presents the bivariate associations between full immunisation and selected socioeconomic and demographic factors in Tamil Nadu and Nagaland, based on the Rao-Scott adjusted chi-square test (reported as adjusted F statistics).

**Table 2:** Distribution of full immunisation status across socioeconomic and demographic characteristics in Tamil Nadu and Nagaland, with Rao-Scott adjusted chi-square tests

Variables	Categories	Nagaland Fully Immunised (%)	Nagaland Not Fully Immunised (%)	χ² (*p*-value)	Tamil Nadu Fully Immunised (%)	Tamil Nadu Not Fully Immunised (%)	χ² (*p*-value)
Maternal education	No education	39.2	60.8	**3.64 (0.014)**	95.7	4.3	**3.08 (0.030)**
	Primary	62.0	38.0		95.5	4.5	
	Secondary	57.3	42.7		91.0	9.0	
	Higher	74.7	25.3		86.4	13.6	
Wealth index	Poor	53.3	46.7	**2.94 (0.046)**	90.1	9.9	1.77 (0.171)
	Middle	63.5	36.5		91.7	8.3	
	Rich	69.6	30.4		87.8	12.2	
Residence	Urban	68.9	31.1	**3.47 (0.034)**	86.4	13.6	**6.81 (0.009)**
	Rural	53.7	46.3		91.7	8.3	
Religion	Hindu	70.6	29.4	0.66 (0.418)	88.5	11.5	**9.68 (0.002)**
	Others	57.3	42.7		96.6	3.4	
Birth order	1	63.2	36.8	**3.72 (0.025)**	87.6	12.4	1.56 (0.212)
	2	61.8	38.2		90.7	9.3	
	≥ 3	48.8	51.2		91.4	8.6	
Distance to facility	No problem	61.7	38.3	**3.18 (0.046)**	88.9	11.1	0.47 (0.493)
	Big problem	52.0	48.0		90.6	9.4	
Sex of child	Male	59.5	40.5	0.36 (0.549)	89.6	10.4	0.19 (0.663)
	Female	56.2	43.8		88.7	11.3	
Mother’s age group	18–24	56.5	43.5	1.59 (0.205)	87.9	12.1	0.92 (0.400)
	25–34	60.6	39.4		89.5	10.5	
	≥ 35	48.0	52.0		94.0	6.0	
Mother’s age in years	Mean ± SD	29.13 ± 5.66	28.42 ± 4.98		26.69 ± 4.54	26.54 ± 4.42	

χ²: adjusted F value for Rao-Scott adjusted chi-square; bold: statistically significant

In Nagaland, full immunisation was significantly associated with maternal education (adjusted F = 3.64, *p* = 0.014), household wealth index (adjusted F = 2.94, *p* = 0.046), place of residence (adjusted F = 3.47, *p* = 0.034), birth order (adjusted F = 3.72, *p* = 0.025), and perceived distance to a health facility (adjusted F = 3.18, *p* = 0.046).

In Tamil Nadu, fewer factors were associated with immunisation status. Maternal education (adjusted F = 3.08, *p* = 0.030), place of residence (adjusted F = 6.81, *p* = 0.009), and religion (adjusted F = 9.68, *p* = 0.002) showed statistically significant associations, while wealth index, birth order, distance to health facility, and maternal age were not significantly associated (*p* > 0.05).

Across both states, no statistically significant association was observed between child sex and immunisation status, indicating no evidence of gender-based disparity in vaccination coverage.

Sensitivity analyses restricted to children with vaccination cards showed broadly similar patterns of association across variables. However, effect estimates were generally stronger, consistent with the higher coverage levels observed in the card-only sample and the potential influence of selection bias (Supplementary Tables 1&2).

Overall, maternal education emerged as a consistent factor associated with full immunisation across both settings, while socioeconomic and geographic disparities were more pronounced in Nagaland than in Tamil Nadu.

### Predictors of immunisation coverage in each state (binary logistic regression)

The multivariable logistic regression results (Table 3) identified factors associated with immunisation status in both states, with the model estimating the odds of being not fully immunised. Maternal education was a consistent predictor of immunisation in both Nagaland (*p* = 0.003) and Tamil Nadu (*p* = 0.029). In both settings, children of mothers with no education had higher odds of being not fully immunised compared to those with higher education (Nagaland: AOR = 2.61; 95% CI: 1.86–3.96; Tamil Nadu: AOR = 2.32; 95% CI: 1.04–2.89). In addition, distance to a health facility was significantly associated with immunisation in Nagaland (*p* = 0.034), whereas in Tamil Nadu, place of residence (AOR = 1.83; 95% CI: 1.17–2.86; *p* = 0.008) and religion (AOR = 4.00; 95% CI: 1.59–10.05; *p* = 0.003) were significant predictors. Other variables, including wealth index, birth order, sex of the child, and maternal age, were not significantly associated with immunisation status after adjustment.

**Table 3:** Summary of binary logistic regression table in both States

Variables (comparison)	Nagaland AOR (95% CI)	*p*-value	Tamil Nadu AOR (95% CI)	*p*-value
Maternal education		0.003		0.029
No education vs. Higher	2.61 (1.86–3.96)		2.32 (1.04–2.89)	
Primary vs. Higher	1.07 (0.32–3.64)		1.32 (0.09–1.92)	
Secondary vs. Higher	1.57 (0.57–4.29)		1.63 (1.10–3.41)	
Wealth index		0.871		0.704
Poor vs. Rich	1.19 (0.58–2.41)		1.21 (0.65–2.26)	
Middle vs. Rich	1.04 (0.50–2.16)		0.91 (0.56–1.49)	
Place of residence		0.369		**0.008**
Urban vs. Rural	1.71 (1.33–1.91)		1.83 (1.17–2.86)	
Religion				**0.003**
Hindu vs. Others	Not significant		4.00 (1.59–10.05)	
Birth order		**0.050**		0.804
1 vs. ≥ 3	0.72 (0.43–1.19)		0.92 (0.41–2.08)	
2 vs. ≥ 3	0.71 (0.43–1.17)		0.82 (0.38–1.78)	
Distance to health facility		**0.034**		0.878
Big problem vs. No problem	1.30 (0.85–1.98)		1.05 (0.59–1.86)	
Sex of child		0.689		0.609
Male vs. Female	0.91 (0.56–1.46)		0.90 (0.59–1.36)	
Mother’s age group		0.759		0.314
18–24 vs. ≥ 35	0.91 (0.44–1.89)		2.18 (0.61–7.84)	
25–34 vs. ≥ 35	0.81 (0.45–1.46)		1.64 (0.48–5.64)	

*CI* Confidence Interval, *AOR* Adjusted Odds Ratio, Bold: statistically significant

**Fig. 4:**
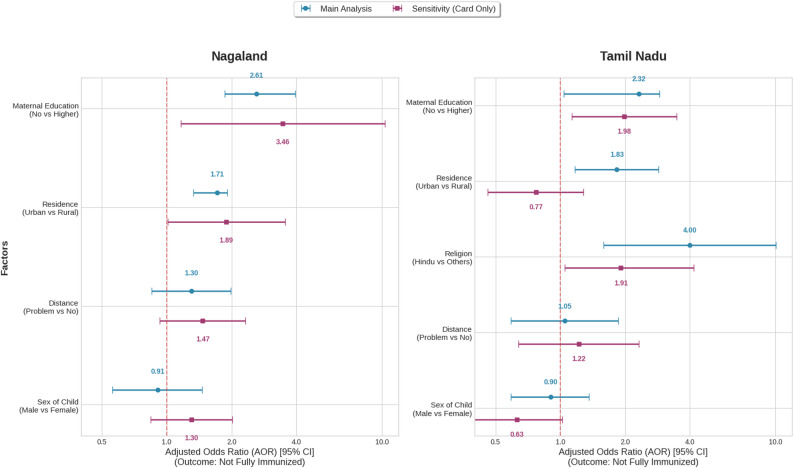
Comparative forest plot of main vs. sensitivity analysis of Immunisation determinants

Our sensitivity analysis restricted to children with vaccination cards showed similar directions of association across predictors confirming the robustness of our findings (Fig. 4). However, effect sizes were generally larger, consistent with higher coverage estimates in the restricted sample and the potential influence of selection bias.

## Discussion

Our study revealed substantial disparities in immunisation coverage between Tamil Nadu and Nagaland, highlighting the influence of socioeconomic and contextual factors on vaccine uptake. Maternal education emerged as a consistent determinant in both states. Children of mothers with no formal education had higher odds of being not fully immunised compared to those with higher education, suggesting higher risk among less educated mothers. This finding is consistent with previous studies such as Vikram et al. (2012), Goodman et al. (2023), and Jejaw et al. (2025), which demonstrate that educated mothers are more likely to understand the benefits of vaccination and effectively engage with health services [5, 10, 12]. Similar associations have been reported in other low- and middle-income settings, including studies from Sub-Saharan Africa and China, where maternal education plays a central role in improving child health service utilisation [19–21]. Strengthening female education and health literacy may therefore contribute to reducing incomplete immunisation, particularly in comparatively lower-coverage settings such as Nagaland.

Wealth-related disparities were more pronounced in Nagaland than in Tamil Nadu. In Nagaland, household wealth was significantly associated with immunisation status at the bivariate level, suggesting that children from economically disadvantaged households remain more vulnerable to incomplete immunisation. In contrast, wealth was not significantly associated with immunisation in Tamil Nadu before and also after adjustment, indicating a more equitable distribution of services. These findings align with previous evidence showing that financial and structural barriers are more influential in settings with weaker health infrastructure [11, 13]. Similar patterns have been observed in low-resource settings in Africa and South Asia, where targeted outreach and community-based strategies have helped reduce wealth-related inequalities in immunisation coverage [19, 20].

Geographic accessibility also emerged as an important structural factor in Nagaland. Children from households that did not perceive distance to a health facility as a major problem had higher immunisation coverage at the bivariate level, although this association was attenuated after adjustment. This suggests that geographic barriers may interact with other socioeconomic and contextual factors rather than acting independently.

In contrast, distance was not significantly associated with immunisation in Tamil Nadu, likely reflecting the state’s relatively strong primary healthcare system and outreach services. Previous studies have similarly shown that limited physical access to health facilities reduces vaccination uptake in remote and underserved areas [1, 11].

However, in the context of Nagaland, sociocultural dynamics may partly explain the weaker independent effect of perceived distance. Many communities are composed of indigenous tribal groups with strong social cohesion, established community support systems, and familiarity with difficult terrain, which may reduce the perceived burden of travel. In addition, community-based delivery approaches, particularly the involvement of frontline health workers and localized outreach strategies may mitigate geographic constraints by bringing services closer to households. These findings suggest that perceived distance may not fully capture access barriers in such settings, where social organization and community-level health systems play a critical role in shaping service utilisation.

Place of residence further highlighted contextual differences between the two states. In Nagaland, urban-rural disparities were evident at the bivariate level, with lower immunisation coverage observed in rural areas, although this association was not statistically significant after adjustment. In contrast, in Tamil Nadu, place of residence remained significantly associated with immunisation status, with urban children having higher odds of being not fully immunised compared to rural children. This pattern may reflect the relatively strong performance of rural health services in Tamil Nadu, where high institutional delivery rates and well-established outreach systems have reduced traditional urban-rural gaps. Similar findings have been reported in parts of India where rural immunisation performance has improved due to targeted public health interventions [4, 8]. However, this may reflect urban slum populations or migration-related disparities.

Birth order was significantly associated with immunisation in Nagaland at the bivariate level, with lower coverage observed among children of higher birth order, although this association did not remain significant after adjustment. This pattern is consistent with previous studies linking higher parity to reduced healthcare utilisation due to competing household demands and resource constraints [14, 15]. No significant association was observed in Tamil Nadu, suggesting that such household-level constraints may be less influential in settings with stronger health system support.

Religion was significantly associated with immunisation in Tamil Nadu, with children from Hindu households having higher odds of being not fully immunised compared to other groups, while no significant association was observed in Nagaland after adjustment. These findings suggest that the influence of cultural and religious factors on immunisation may vary by regional context. Previous studies have similarly reported that the relationship between religion and vaccination is context-specific and mediated by local beliefs and social norms [8, 16]. Engagement with community and faith leaders may therefore be important in improving vaccine acceptance in diverse settings.

No significant association was observed between child sex and immunisation status in either state, indicating no evidence of gender-based disparities. This reflects progress toward gender equity in child health interventions and contrasts with earlier findings in some parts of India [8]. Maternal age was not significantly associated with immunisation after adjustment in either state.

Beyond structural access and socioeconomic factors, continuity of interaction with the health system and the quality of documentation may also influence immunisation outcomes. The variations in documentation and continuity of care may also contribute to differences in observed immunisation coverage. Access to vaccination records, including immunisation cards, is closely linked to interactions with the health system and can influence both the accuracy of reported coverage and follow-up for subsequent doses. In settings where health system contact is less consistent, gaps in documentation may reflect broader challenges in service utilisation and continuity of care. However, these factors were not directly assessed in the present study and should be interpreted with caution. Moreover, differences in state-level health system governance, service delivery efficiency, and program implementation may also contribute to the observed disparities.

Overall, the findings suggest that while Tamil Nadu’s relatively strong and equitable primary healthcare system has reduced socioeconomic gradients in immunisation, disparities in Nagaland are more strongly influenced by educational, economic, and structural factors. Addressing these gaps will require context-specific strategies, including strengthening female education, improving service accessibility in underserved areas, and expanding community-based outreach programmes. Similar strategies to those observed in high-performing settings such as Tamil Nadu have been implemented in other low- and middle-income countries (LMICs), including strengthened primary healthcare systems, community health worker engagement, and targeted outreach to underserved populations [23]. Evidence from LMICs highlights the importance of community-based delivery models and maternal education in improving immunisation uptake in socioeconomically constrained settings [22]. Government-led initiatives such as Intensified Mission Indradhanush (IMI), which focus on reaching underserved and high-risk populations through targeted campaigns, have played a critical role in improving immunisation coverage in India. Such strategies may offer important lessons for states with lower coverage, particularly in addressing geographic and socioeconomic disparities [18].

### Strengths and limitations

#### Strengths

Our study used a large, representative dataset from NFHS-5; comprehensive range of variables. The focus on socioeconomic and demographic determinants, such as maternal education, wealth index, and healthcare access, offers a multidimensional perspective.

#### Limitations

The cross-sectional design of NFHS-5 limits causal interpretation of the observed associations. Vaccination status partly relies on maternal recall, which may introduce recall bias and misclassification, particularly in the absence of vaccination cards. In addition, residual confounding from unmeasured factors such as health system performance, cultural practices, and local service delivery variations cannot be ruled out. Finally, the use of perceived distance as a proxy for geographic access may not fully capture actual accessibility to health services, especially in geographically diverse settings.

Further studies should focus on conducting longitudinal studies to assess the long-term impact of maternal education and socioeconomic improvements on Immunisation rates, particularly in low-performing regions like Nagaland, and eventually investigate the role of health system infrastructure and human resource availability in influencing Immunisation coverage.

## Conclusion

Our study demonstrates that disparities in child immunisation coverage between Tamil Nadu and Nagaland reflect underlying socioeconomic and structural differences in access to and utilisation of health services. Maternal education was consistently associated with immunisation status in both states, with children of less educated mothers more likely to be incompletely immunised, highlighting the important role of female education in shaping preventive health behaviours.

In Nagaland, lower immunisation coverage appears to be influenced by a combination of socioeconomic disadvantage and structural constraints, including wealth-related inequalities and geographic access barriers observed at the bivariate level. In contrast, Tamil Nadu’s high coverage and fewer socioeconomic differentials suggest a more equitable delivery of immunisation services, supported by a strong primary healthcare system and effective outreach mechanisms.

These findings indicate that achieving equitable immunisation coverage requires more than the availability of vaccines alone. Context-specific strategies are needed, including strengthening female education, improving access to services in underserved areas, and reinforcing community-based outreach efforts. Addressing these interconnected determinants will be essential for advancing progress toward universal immunisation coverage in India and reducing preventable childhood morbidity.

## Supplementary Information

Supplementary Material 1.

## Data Availability

The data analysed for this study are publicly available through the Demographic and Health Surveys (DHS) Program. Specifically, this study utilized the dataset from the National Family Health Survey (NFHS-5, 2019-2021) for Tamil Nadu and Nagaland, which can be accessed from the DHS website ( [https://dhsprogram.com/data/available-datasets.cfm](https:/dhsprogram.com/data/available-datasets.cfm) ) upon approval of a data request. All analyses and findings presented in this article are based on secondary data and comply with ethical research practices.
