# Supplementary material for: Bridging the immunisation gap: socioeconomic and geographic drivers of pediatric immunisation disparities between different Indian states
Source: BMC Pediatr. 2026 Apr 14;26:483. doi: 10.1186/s12887-026-06822-6 (PMC13202825; doi:10.1186/s12887-026-06822-6)
Supplement: Supplementary file 1 — Supplementary Material 1. [file 12887_2026_6822_MOESM1_ESM.docx]

**SUPPLEMENTARY FILES**

Supplementary table 1. Distribution children according to socioeconomic and demographic variables by immunization status, Tamil Nadu and Nagaland (NFHS-5, 2019-21)

| **Variable** | **Category** | **Fully immunized (%) - Nagaland** | **Not Fully immunized (%) - Nagaland** | **Fully immunized (%) - Tamil Nadu** | **Not Fully immunized (%) - Tamil Nadu** |
| --- | --- | --- | --- | --- | --- |
| **Type of residence** | Urban | 66.6 | 33.4 | 47.4 | 52.6 |
|  | Rural | 44.8 | 55.2 | 52.5 | 47.5 |
| **Mother’s age (years)** | Mean ± SD | 28.0 ± 5.14 | 29.0 ± 5.69 | 26.82 ± 4.52 | 26.11 ± 3.48 |
| **Highest educational level** | No education | 32.6 | 67.4 | 64.9 | 35.1 |
|  | Primary | 62.6 | 37.4 | 75.9 | 24.1 |
|  | Secondary | 49.4 | 50.6 | 57.9 | 42.1 |
|  | Higher | 60.1 | 39.9 | 41.8 | 58.2 |
| **Wealth index** | Poor | 45.2 | 54.8 | 52.3 | 47.7 |
|  | Middle | 53.7 | 46.3 | 52.4 | 47.6 |
|  | Rich | 67.7 | 32.3 | 48.5 | 51.5 |
| **Distance to health facility** | No problem | 55.2 | 44.8 | 50.1 | 49.9 |
|  | Big problem | 43.0 | 57.0 | 49.2 | 50.8 |
| **Religion** | Hindu | 78.3 | 21.7 | 48.2 | 51.8 |
|  | Christian | 49.4 | 50.6 | 80.3 | 19.7 |
|  | Muslim | 28.1 | 71.9 | 75.5 | 24.5 |
| **Birth order** | 1 | 57.9 | 42.1 | 45.8 | 54.2 |
|  | 2 | 55.1 | 44.9 | 57.1 | 42.9 |
|  | 3 | 41.1 | 58.9 | 47.4 | 52.6 |
|  | 4 or more | 37.9 | 62.1 | 47.8 | 52.2 |
| **Sex of child** | Male | 52.4 | 47.6 | 45.3 | 54.7 |
|  | Female | 47.7 | 52.3 | 56.7 | 43.3 |

Supplementary table 2: Chi-Square (χ²) test of association between immunization status and selected variables, Tamil Nadu and Nagaland

| **Variable** | **χ² (p-value) - Nagaland** | **χ² (p-value) - Tamil Nadu** |
| --- | --- | --- |
| Type of residence | 13.011 (p = 0.001) | 0.780 (p = 0.377) |
| Mother’s age (years) | 3.501 (p = 0.598) | 11.463 (p = 0.113) |
| Highest educational level | 3.983 (p = 0.006) | 9.764 (p = 0.024) |
| Wealth index | 9.702 (p = 0.011) | 0.431 (p = 0.808) |
| Distance to health facility | 5.550 (p = 0.018) | 0.014 (p = 0.906) |
| Religion | 5.540 (p = 0.038) | 5.609 (p = 0.244) |
| Birth order | 20.504 (p = 0.012) | 3.648 (p = 0.488) |
| Sex of child | 0.861 (  p = 0.353) | 3.665 (p = 0.058) |

**Note:** χ² = Chi-square test statistic.

Figure 1: Mother's education level distribution per states

Figure 2: immunization coverage distribution by State


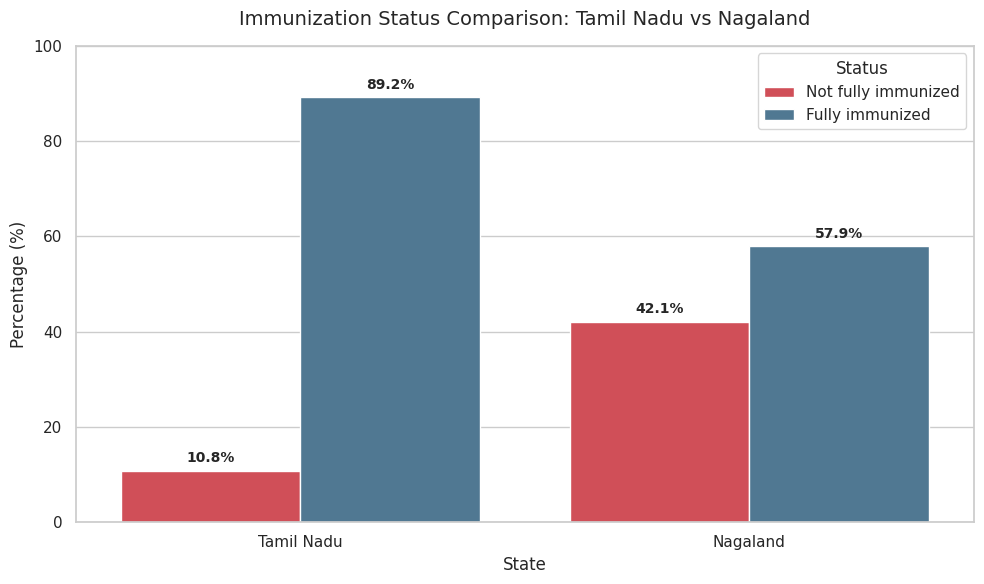


Figure 3: immunization coverage distribution by States


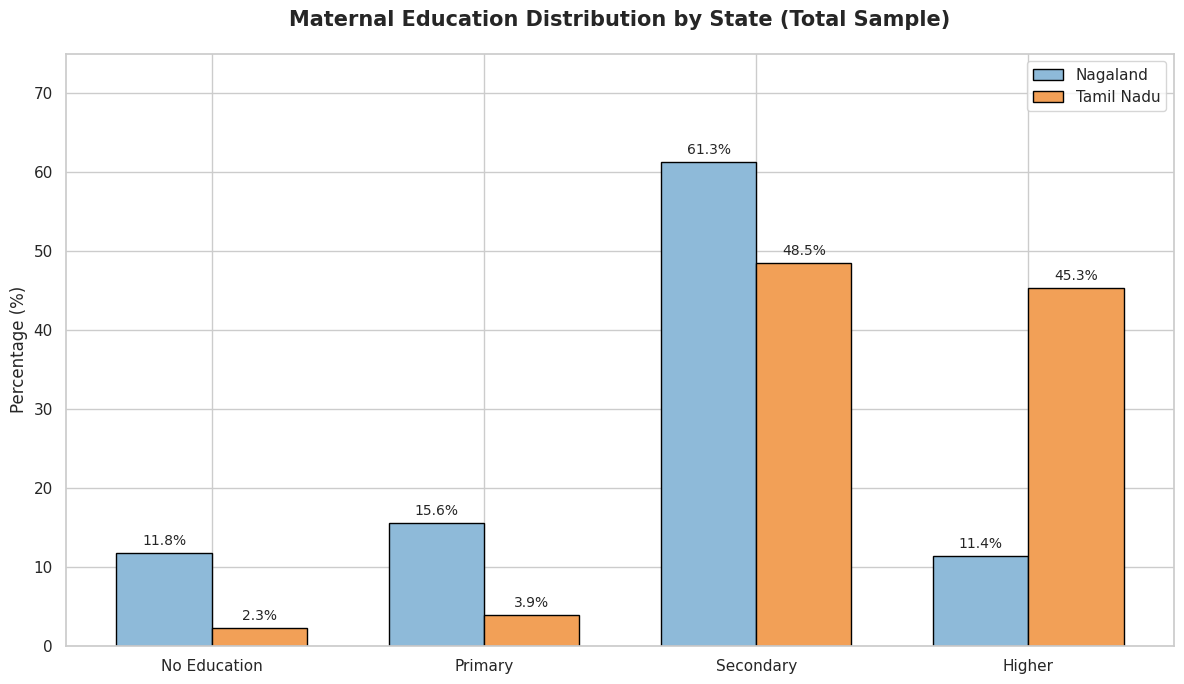


Figure 4: immunization coverage distribution by States

Supplementary table 3. Assessment of multicollinearity among independent variables

| **Variables** | **Tolerance** | **VIF** |
| --- | --- | --- |
| Sex of child | 0.995 | 1.01 |
| Distance to health facility | 0.892 | 1.12 |
| Birth order | 0.779 | 1.28 |
| Religion | 0.805 | 1.24 |
| Place of residence | 0.809 | 1.24 |
| Household wealth index | 0.581 | 1.72 |
| Maternal education | 0.673 | 1.49 |
| Mother's age group | 0.863 | 1.16 |

*Note: Tolerance and variance inflation factor (VIF) were used to assess multicollinearity. A VIF < 2 and tolerance > 0.1 indicate no evidence of multicollinearity.*

Supplementary table 4: Summary of binary logistic regression table in both States

| **Variable** | **AOR (95% CI) Tamil Nadu** | **p-value** | **AOR (95% CI) Nagaland** | **p-value** |
| --- | --- | --- | --- | --- |
| **Type of residence** (Urban vs Rural) | 0.77 (0.46-1.28) | 0.309 | 1.89 (1.01-3.54) | 0.047 |
| **Religion** | Not significant | 0.389 | Not significant | 0.162 |
| **Distance to health facility** (Big problem vs No problem) | 1.22 (0.64-2.32) | 0.546 | 1.47 (0.93-2.32) | 0.096 |
| **Highest educational level** (Higher vs No education) | 1.98 (1.13-3.46) | 0.017 | 3.46 (1.16-10.30) | 0.026 |
| **Birth order number** (≥3 vs 1-2) | Not significant | 0.600 | Not significant | 0.500 |
| **Sex of child** (Male vs Female) | 0.63 (0.39-1.02) | 0.061 | 1.30 (0.84-2.01) | 0.243 |
| **Mother’s age (years)** | 1.22 (1.01-1.46) | 0.037 | 1.01 (0.85-1.19) | 0.925 |
| **Wealth index** (Rich vs Poor) | Not significant | 0.860 | Not significant | 0.373 |
| **Age group of mothers** | Not significant | 0.078 | Not significant | 0.789 |
